# Effectiveness of Genetic Counseling Modalities on Knowledge, Attitudes, and Premarital Screening Intentions for Thalassemia Prevention: A Multi-Site Three-Arm Randomized Controlled Trial

**DOI:** 10.3390/ijerph23070914

**Published:** 2026-07-16

**Authors:** Henri Setiawan, Chengyang Li, Muhammad Kamil Che Hasan, Thandar Soe @ Sumaiyah Jamaludin, Yudisa Diaz Lutfi Sandi, Andan Firmansyah, Yuyun Rahayu, Ima Sukmawati, Lilis Lismayanti, Edwin Nugroho Njoto

**Affiliations:** 1 Faculty of Medicine and Health, Institut Teknologi Sepuluh Nopember, Surabaya 60111, Indonesia; henrisetiawan1989@gmail.com; 2 School of Nursing, Fujian Medical University, Fuzhou 350005, China; 18750905518@163.com; 3 Kulliyyah of Nursing, International Islamic University Malaysia, Kuantan 25200, Malaysia; mkamil@iium.edu.my (M.K.C.H.); sumaiyah@iium.edu.my (T.S.@S.J.); 4 Department of Nursing, Politeknik Negeri Subang, Subang 41285, Indonesia; yudisadiaz@gmail.com; 5 Department of Nursing, STIKes Muhammadiyah Ciamis, Ciamis 46216, Indonesia; andan.rock@gmail.com (A.F.); yuyunwahyu32@gmail.com (Y.R.); imasukma90@gmail.com (I.S.); lismayantililis123@gmail.com (L.L.)

**Keywords:** thalassemia prevention, genetic counseling, premarital screening, digital health education, educational video, digital leaflet, screening intention

## Abstract

**Highlights:**

**Public health relevance—How does this work relate to a public health issue?**

- Thalassemia remains a major hereditary health problem in many low- and middle-income countries, including Indonesia, where premarital screening uptake remains limited.
- Improving genetic counseling accessibility among prospective couples is essential to support early prevention and informed reproductive decision-making.

**Public health significance—Why is this work of significance to public health?**

- Digitally delivered genetic counseling was associated with greater improvements in knowledge, attitudes, and screening intention than face-to-face counseling within the study setting.
- The findings highlight the potential of digital counseling approaches to strengthen premarital thalassemia prevention strategies in resource-limited and community-based healthcare settings.

**Public health implications—What are the key implications or messages for practitioners, policy makers and/or researchers in public health?**

- Integrating digital genetic counseling into premarital health services may improve the reach, consistency, and accessibility of thalassemia prevention programs.
- Future reproductive health interventions should combine digital education with culturally responsive counseling and supportive community engagement to address sociocultural barriers to premarital screening participation.

**Abstract:**

Thalassemia prevention relies on effective premarital screening. However, participation remains suboptimal because of multiple individual, sociocultural, and structural barriers, including limited knowledge, stigma, cultural and religious beliefs, healthcare accessibility, and socioeconomic constraints. This multi-site three-arm randomized controlled trial evaluated the effectiveness of digital leaflet-based, educational video-based, and face-to-face genetic counseling on knowledge, attitudes, and screening intentions among prospective couples in Ciamis District, Indonesia. A total of 177 prospective couples were randomized (digital leaflet = 59, video = 59, face-to-face = 59). After follow-up, 41, 53, and 59 participants, respectively, completed the post-intervention assessment and were included in the final analysis. Outcomes were measured at baseline and post-intervention using validated questionnaires and analyzed using Wilcoxon signed-rank tests and repeated-measures analysis of variance. Digital leaflet-based counseling significantly improved knowledge and screening intention (*p* < 0.001), while educational video-based counseling significantly improved knowledge, attitudes, and intention (*p* < 0.05). Face-to-face counseling showed no statistically significant changes across outcomes. Between-group analyses revealed significant differences in knowledge (η^2^ = 0.059, *p* < 0.001), attitudes (η^2^ = 0.080, *p* < 0.001), and intentions (η^2^ = 0.084, *p* < 0.001), with digital interventions outperforming face-to-face counseling. These findings suggest that digital genetic counseling modalities were associated with greater improvements in knowledge, attitudes, and premarital screening intentions than face-to-face counseling in this study setting.

## 1. Introduction

Thalassemia is one of the most prevalent inherited hemoglobin disorders worldwide and continues to pose a substantial public health burden, particularly in low- and middle-income countries, including Indonesia. The number of thalassemia sufferers in Indonesia increased from 4896 in 2012 to 8761 in 2018. The latest data in 2019 shows a significant increase to 10,500 patients. This data is predicted to continue to grow, with as many as 1500 new cases diagnosed each year [1,2,3]. The disease requires lifelong management through regular blood transfusions and iron chelation therapy, resulting in considerable physical, psychological, social, and economic consequences for affected individuals and their families [3,4]. Due to its autosomal recessive inheritance pattern, preventive strategies focusing on carrier identification and premarital screening have become essential approaches to reducing the incidence of severe thalassemia births. However, despite the recognized importance of premarital screening programs, participation rates among reproductive-age populations remain relatively low in many settings, indicating persistent challenges in preventive awareness and screening acceptance [5].

Knowledge and attitudes toward hereditary diseases play a crucial role in shaping individuals’ willingness to participate in premarital screening programs. Previous studies have demonstrated that inadequate understanding of thalassemia inheritance, misconceptions regarding genetic testing, fear of social stigma, and concerns about marital consequences contribute to low screening uptake among prospective couples. Positive attitudes and sufficient knowledge are strongly associated with preventive decision-making and screening participation [6,7]. Nevertheless, existing educational efforts in many communities remain fragmented and inconsistent, limiting their ability to effectively influence behavioral intentions toward premarital thalassemia screening [8].

Genetic counseling has been widely recommended as an important strategy to improve public understanding of hereditary diseases and support informed reproductive decision-making. Conventional face-to-face counseling enables interactive communication and individualized education, allowing participants to directly discuss concerns related to genetic risk and screening outcomes [9,10]. Despite these advantages, direct counseling approaches often require substantial healthcare resources, trained personnel, and adequate consultation time, which may limit scalability and accessibility in community-based prevention programs [11,12]. These challenges create an important need for alternative counseling models that are more efficient, accessible, and adaptable to larger populations.

The rapid development of digital health education has introduced new opportunities for delivering genetic counseling through multimedia-based interventions, including digital leaflets and educational videos. Digital educational approaches may provide broader dissemination, standardized information delivery, lower implementation costs, and greater accessibility for young adults who are highly engaged with digital media platforms. Several studies in public health have reported that audiovisual and digital educational interventions can improve health literacy and preventive behaviors [13,14,15,16]. However, evidence regarding the effectiveness of digital counseling modalities specifically for thalassemia prevention remains limited. Compared with conventional face-to-face counseling, digital interventions may facilitate repeated access to educational materials and ensure greater consistency in information delivery, whereas face-to-face counseling offers personalized interaction and immediate clarification of participants’ concerns. These differences in delivery characteristics may influence learning outcomes and behavioral intentions through distinct mechanisms, highlighting the need for direct comparative evaluation of different genetic counseling modalities [10,11].

In addition, most previous studies related to thalassemia education have predominantly evaluated improvements in knowledge as the principal outcome [17,18,19], whereas psychosocial determinants of preventive behavior, particularly attitudes and intentions toward premarital screening, have received considerably less attention despite their recognized role in influencing health-related decision-making. Moreover, the available evidence has largely been generated from cross-sectional or quasi-experimental studies, limiting the ability to establish causal relationships between educational interventions and behavioral outcomes [20,21,22]. Consequently, it remains unclear whether the observed benefits are attributable to the educational content itself or to the mode of intervention delivery. This evidence gap is particularly relevant in Southeast Asian settings, where the burden of thalassemia is high but comparative evidence regarding scalable counseling strategies for prospective couples remains limited.

Prospective couples represent a strategically important target population for thalassemia prevention because reproductive and marital decisions are commonly established before marriage. Identifying effective counseling approaches for this population is therefore essential to strengthen premarital screening programs and support early preventive decision-making [23,24]. Nevertheless, there is currently insufficient evidence regarding which counseling modality is most effective in improving knowledge, attitudes, and intentions toward premarital thalassemia screening among engaged couples.

Therefore, this multi-site three-arm randomized controlled trial was designed to directly compare three genetic counseling modalities (digital leaflets, educational videos, and face-to-face counseling) using identical educational content to determine whether the mode of delivery differentially influences knowledge, attitudes, and premarital screening intentions among prospective couples.

## 2. Materials and Methods

### 2.1. Study Design

This study was designed as a multi-site, three-arm randomized controlled trial involving prospective couples as the target population. Participants were allocated to one of three intervention groups: digital leaflet-based genetic counseling, educational video-based genetic counseling, or conventional face-to-face genetic counseling. The study was conducted in accordance with the Consolidated Standards of Reporting Trials (CONSORT) guidelines. It represents an extension of a previously registered trial in the Chinese Clinical Trial Registry (ChiCTR; Registration No. ChiCTR2100049918; registered on 10 August 2021; public registry record: https://www.chictr.org.cn/showproj.html?proj=129504 accessed date on 30 July 2024). Because the present study differs from the original trial in terms of the study setting, participant population, and outcome measures, a new registration was submitted to the Chinese Clinical Trial Registry on 1 February 2026 (Registration PID: 323456) and was under administrative review at the time of manuscript submission.

### 2.2. Sample Size Calculation

The sample size was calculated for comparing mean differences across three intervention groups using one-way analysis of variance (ANOVA), assuming a medium effect size (Cohen’s f = 0.25), a two-sided significance level of 0.05, and 80% statistical power. The minimum required sample size was estimated at 159 participants (53 per group). To account for potential attrition and ensure balanced allocation, the sample size was increased by approximately 10%, resulting in a target enrollment of 177 participants (59 per group). Although differential attrition occurred during follow-up, particularly in the digital leaflet group, the final analyzed sample remained sufficient to detect medium-sized effects across intervention comparisons.

### 2.3. Participants and Settings

The participants were recruited from the Department of Health Education for Prospective Couples at the Religious Affairs Offices across 15 subdistricts in Ciamis District, West Java Province, Indonesia. Recruitment and data collection were conducted from 15 June 2025 to 14 January 2026, among individuals registered as prospective marriage partners who attended premarital educational programs and marriage administrative services. Although the target population comprised prospective couples, only one member from each couple was enrolled in the study. If both partners met the eligibility criteria, only one individual was invited to participate to ensure the independence of observations and avoid clustering at the couple level. Screening procedures were carried out by the research team to ensure that all participants fulfilled the eligibility criteria prior to enrollment in the study.

Consecutive sampling was employed to recruit all eligible participants during the study period, whereas randomization was performed only after enrollment and baseline assessment to ensure unbiased allocation across the intervention groups. The number of participants recruited from each subdistrict was proportionally adjusted according to the number of prospective couples registered at each Religious Affairs Office during the study period. Eligible participants who attended premarital educational sessions were consecutively approached and invited to participate until the required sample size for each study site was achieved.

The inclusion criteria were:

- Prospective couples registered for premarital administrative procedures at the Religious Affairs Office.
- Aged 18 years or older.
- Able to communicate in the Indonesian language.
- Willing to participate by providing informed consent.
- Having access to a smartphone or digital device to receive the digital educational interventions.

The exclusion criteria included:

- Having previously received formal genetic counseling related to thalassemia or hereditary diseases.
- Being diagnosed with thalassemia or other major hereditary hematologic disorders
- Having cognitive, visual, or hearing impairments that could interfere with participation in the intervention or completion of the questionnaires
- Withdrawing participation during the study period.

### 2.4. Randomization and Allocation Concealment

After eligibility screening and baseline assessment, participants were randomly assigned to one of the three intervention groups in a 1:1:1 allocation ratio: digital leaflet-based genetic counseling, educational video-based genetic counseling, or face-to-face genetic counseling. The random allocation sequence was generated using the web-based randomization platform (https://www.randomizer.org/) by an independent researcher who was not involved in participant recruitment, intervention delivery, or outcome assessment.

Eligible participants were enrolled consecutively and assigned according to the next available allocation in the pre-generated randomization sequence. Thus, treatment allocation followed the computer-generated randomization list rather than a fixed or alternating assignment pattern. This procedure was implemented consistently across all study sites to maintain balanced allocation while preserving the integrity of the randomization process.

Allocation concealment was maintained using sequentially numbered, opaque, sealed envelopes prepared by the independent researcher. The envelopes were opened only after participant eligibility had been confirmed and written informed consent had been obtained. Owing to the nature of the educational interventions, blinding of participants and intervention providers was not feasible. However, outcome assessors and data analysts remained blinded to group allocation to minimize assessment and analytical bias.

### 2.5. Intervention

Participants in this study were randomly assigned to one of three genetic counseling intervention groups: digital leaflet-based counseling, educational video-based counseling, or face-to-face genetic counseling. All interventions were designed to provide standardized information regarding thalassemia prevention, including the definition of thalassemia, inheritance patterns, carrier status, health and reproductive consequences, the importance of premarital screening, and preventive decision-making. To ensure consistency across intervention groups, all educational content was developed based on current thalassemia prevention guidelines and reviewed by healthcare professionals with expertise in genetic counseling and health education.

All interventions were delivered once during the premarital educational session at the participating Religious Affairs Offices. Standardized intervention procedures were implemented across all study centers to maintain intervention fidelity and consistency throughout the trial.

#### 2.5.1. Digital Leaflet-Based Genetic Counseling

Participants in the first intervention group received digital leaflet-based genetic counseling. The intervention consisted of visually designed digital leaflets containing concise educational messages, infographics, illustrations, and simplified explanations related to thalassemia prevention and premarital screening. The leaflet content and visual design were developed and validated in a previous study and have been published elsewhere [25]. The educational materials were designed to ensure clarity, readability, and cultural relevance for reproductive-age populations. The digital leaflets were distributed electronically through smartphone-accessible platforms, allowing participants to review the educational materials repeatedly during the intervention period.

#### 2.5.2. Video-Based Genetic Counseling

Participants in the second intervention group received educational video-based genetic counseling. The intervention utilized a structured educational video combining audiovisual narration, animation, illustrations, and explanatory presentations regarding thalassemia inheritance, carrier screening, reproductive risks, and preventive strategies. The educational video was developed as part of a collaborative research and public health education project between the research team and the Government of Ciamis Regency to support community-based thalassemia prevention initiatives. The video was designed as an openly accessible educational resource and is publicly available through YouTube (https://www.youtube.com/watch?v=WdvhbD_E1wI accessed date on 25 April 2025). The educational video was delivered digitally and designed to improve participant engagement and understanding through simultaneous visual and auditory learning approaches.

#### 2.5.3. Direct Face-to-Face Genetic Counseling

Participants in the third intervention group received direct face-to-face genetic counseling conducted by trained healthcare personnel. The counseling sessions were delivered individually using standardized educational materials and interactive communication methods adapted from a previously published genetic counseling framework [9], with content contextualized to focus specifically on thalassemia prevention, inheritance patterns, carrier screening, reproductive risks, and premarital preventive decision-making. Participants were provided opportunities to ask questions and discuss concerns related to thalassemia screening, hereditary risk, and preventive actions. This intervention emphasized personalized communication and clarification to support informed decision-making regarding premarital screening participation.

The characteristics of the three intervention groups, including delivery mode, session duration, counselor qualifications, intervention fidelity, and standardization procedures, are summarized in Table 1.

### 2.6. Outcomes

The study outcomes consisted of knowledge, attitudes, and intentions toward premarital thalassemia screening. Knowledge and attitudes were measured using an adapted questionnaire previously developed and validated for thalassemia prevention and screening [26]. The knowledge section included 10 items assessing participants’ understanding of thalassemia inheritance, carrier status, diagnosis, treatment, and prevention. Responses were categorized as “true,” “false,” or “do not know,” with scores classified into good knowledge (≥60% correct responses) and poor knowledge (<60% correct responses). Attitudes toward thalassemia prevention were assessed using three dichotomous (yes/no) items evaluating participants’ willingness to undergo premarital screening, donate blood for patients with thalassemia, and disseminate information regarding thalassemia prevention. Attitude scores were categorized into positive attitudes (≥60%) and negative attitudes (<60%). The questionnaire was adapted from a previously published instrument that was developed through literature review, translation verification, pilot testing in a population similar to the target population, and internal consistency reliability assessment before implementation. According to the original instrument development study, the adapted knowledge and attitude questionnaire demonstrated a Cronbach’s alpha coefficient of 0.603, indicating moderate internal consistency [26].

Intentions toward premarital thalassemia screening were measured using a single-item question adapted from previous study, asking participants whether they intended to undergo thalassemia screening [27]. The instrument was directly adopted from previous validated research and therefore was not subjected to additional validity and reliability testing in the present study.

### 2.7. Data Collection and Statistical Analysis

Data collection was conducted from 15 June 2025 to 14 January 2026 at the Department of Health Education for Prospective Couples across 15 Religious Affairs Offices in Ciamis District, West Java Province, Indonesia. After providing written informed consent, eligible participants completed baseline assessments before receiving the assigned intervention. Outcome measurements, including knowledge, attitudes, and intentions toward premarital thalassemia screening, were assessed at baseline (pretest) and after completion of the intervention (posttest). All data were collected using standardized procedures by trained research assistants across participating centers to ensure consistency throughout the study.

The collected data were coded and analyzed using Jamovi software (Version 2.6). Descriptive statistics, including frequencies, percentages, means, and standard deviations, were used to summarize participant characteristics and study variables. Homogeneity of baseline characteristics among intervention groups was examined using Pearson’s chi-square test and contingency coefficients. Prior to inferential analysis, data distribution and normality assumptions were evaluated. Within-group differences between pretest and posttest scores were analyzed using the Wilcoxon signed-rank test because several outcome variables did not meet normality assumptions. Effect sizes for within-group analyses were calculated using the r statistic. Between-group differences over time were evaluated using repeated measures analysis of variance (Repeated Measures ANOVA) to examine intervention group, time, and group-by-time interaction effects. Post hoc comparisons were conducted using Tukey’s test for significant findings, and effect sizes were reported using eta squared (η^2^).

Analyses were conducted using a complete-case approach. Participants who were lost to follow-up completed the baseline assessment but did not complete the post-intervention assessment; consequently, no post-intervention outcome data were available for these participants, and they were excluded from the final analyses. No imputation procedures were applied for missing outcome data. To assess the robustness of the primary findings and account for baseline imbalances among intervention groups, a sensitivity analysis using Generalized Estimating Equations (GEEs) was performed. The GEE models included intervention group, time, and group-by-time interaction terms while adjusting for sex, employment status, and monthly income to account for within-subject correlations and estimate adjusted intervention effects over time.

### 2.8. Ethical Consideration

This study was carried out in accordance with the ethical principles stated in the Declaration of Helsinki. Ethical approval was obtained from the Health Research Ethics Committee of STIKes Muhammadiyah Ciamis under protocol number 148/KEPK-MUCIS/V/2025, dated 1 May 2025. Prior to participation, all participants received explanations regarding the study objectives, procedures, potential benefits, and their rights as research participants, after which written informed consent was obtained. Participant confidentiality and privacy were strictly protected throughout the study process. All collected data were anonymized and stored securely, with access limited only to the research team for academic and research purposes. Participants were also informed that their participation was voluntary and that they had the right to withdraw from the study at any stage without any penalty or consequences.

### 2.9. Generative Artificial Intelligence Disclosure

In this study, Generative Artificial Intelligence (GenAI) tools, specifically OpenAI ChatGPT (GPT-5.5), were used solely to support non-analytical activities, including language refinement, grammar correction, formatting assistance, and improvement of manuscript readability. GenAI was not used for primary data collection, data analysis, statistical interpretation, outcome generation, or scientific decision-making. All study design, methodological decisions, interpretation of findings, and final content were conducted, verified, and approved by the researchers. The research team maintained full responsibility for the accuracy, integrity, and originality of all scientific outputs.

## 3. Results

### 3.1. Recruitment and Characteristics of Participants

**Figure 1:**
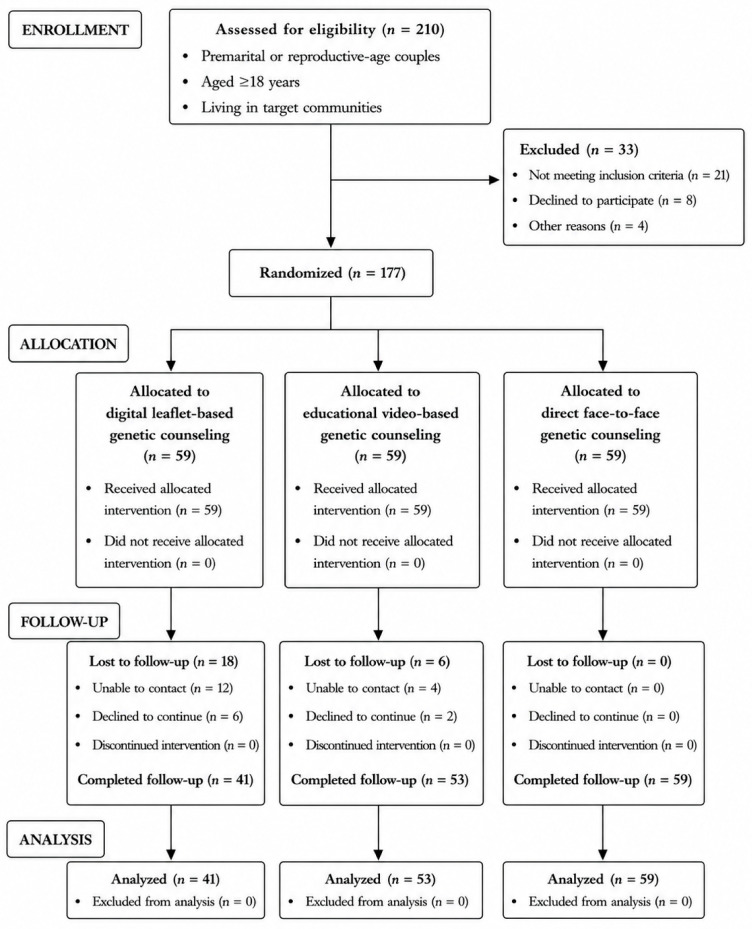
CONSORT flow diagram of participant enrollment, randomization, allocation, follow-up, and analysis.

A total of 210 prospective couples were screened for eligibility across 15 Religious Affairs Offices in Ciamis District, West Java Province, Indonesia. Following the screening process, 33 individuals were excluded because they did not meet the inclusion criteria, declined participation, or were excluded for other reasons. Subsequently, 177 participants were randomly allocated into three intervention groups with an equal allocation ratio of 1:1:1, consisting of digital leaflet-based genetic counseling (*n* = 59), educational video-based genetic counseling (*n* = 59), and direct face-to-face genetic counseling (*n* = 59). Participant attrition occurred during the follow-up phase in two intervention groups. The digital leaflet group experienced the highest loss to follow-up, with 18 participants not completing the study, resulting in 41 participants included in the final analysis. In the educational video group, six participants were lost during follow-up, leaving 53 participants for analysis. All participants allocated to the direct genetic counseling group completed the study and were included in the final analysis (*n* = 59). The detailed information regarding the participants is presented in Figure 1.

Baseline characteristics of the participants are presented in Table 2. The majority of participants were aged below the mean age of 23.4 years (54.2%), female (61.4%), had completed senior high school education (60.1%), and were unemployed (60.1%). More than half of the participants reported a monthly income below US$127 (60.8%). Baseline measurements further indicated that most participants demonstrated poor knowledge regarding thalassemia prevention (57.5%), whereas positive attitudes and intentions toward premarital thalassemia screening were observed in 62.7% and 63.4% of participants, respectively. Homogeneity testing demonstrated no statistically significant differences among intervention groups with respect to age and educational level (*p* > 0.05). However, statistically significant differences were identified for sex, employment status, monthly income, knowledge, attitudes, and screening intentions across the intervention groups (*p* < 0.05).

### 3.2. Within-Group Effects of the Intervention on Participants’ Outcomes

**Figure 2:**
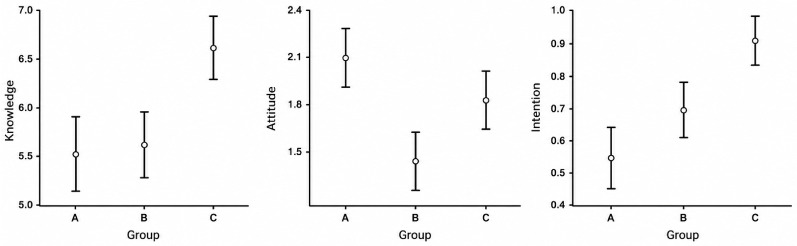
Mean scores of outcomes across groups.

The Wilcoxon signed-rank test showed that digital leaflet–based genetic counseling significantly improved knowledge (Z = −5.916, *p* < 0.001, r = 0.92) and intention (Z = −6.164, *p* < 0.001, r = 0.96), but did not significantly change attitude (Z = −1.633, *p* = 0.102, r = 0.25). Educational video–based genetic counseling also produced significant improvements in knowledge (Z = −5.488, *p* < 0.001, r = 0.75) and attitude (Z = −3.800, *p* < 0.001, r = 0.52), while intention also showed a significant change (Z = −2.837, *p* = 0.005, r = 0.39), although with a smaller effect size. In contrast, face-to-face genetic counseling did not produce statistically significant changes in knowledge (Z = −1.000, *p* = 0.317, r = 0.13), attitude (Z = −0.378, *p* = 0.705, r = 0.05), or intention (Z = −0.378, *p* = 0.705, r = 0.05). These patterns are visually reflected in Figure 2, where higher post-intervention mean scores are observed in the digitally delivered intervention groups, particularly for knowledge and intention in the digital leaflet–based counseling group and attitude in the educational video–based counseling group, whereas the face-to-face group demonstrates comparatively smaller changes. Detailed statistical results are presented in Table 3.

### 3.3. Between-Group Effects of the Intervention on Participants’ Outcomes

**Figure 3:**
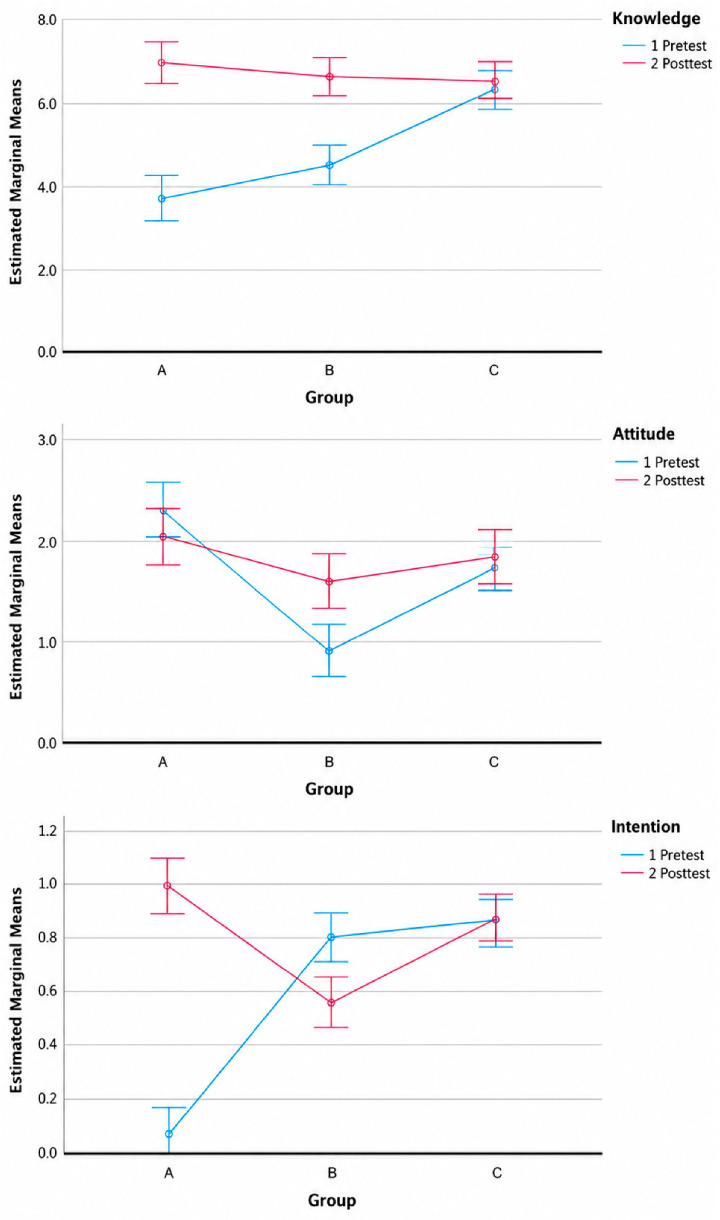
Estimated marginal means of knowledge, attitude, and intention scores across intervention groups at pretest and posttest. A = Digital leaflet-based genetic counseling (*n* = 41); B = educational video-based genetic counseling (*n* = 53); C = direct face-to-face genetic counseling (*n* = 59).

Table 4 shows significant between-group differences in participants’ knowledge (η^2^ = 0.059, *p* < 0.001), attitude (η^2^ = 0.080, *p* < 0.001), and intention (η^2^ = 0.084, *p* < 0.001) outcomes following the intervention. Post hoc Tukey analyses demonstrated significant differences in knowledge scores between groups A and C (MD = −1.047, *p* < 0.001) and groups B and C (MD = —0.937, *p* < 0.001), whereas no significant difference was identified between groups A and B (*p* = 0.883). For attitude outcomes, significant differences were found between groups A and B (MD = 0.691, *p* < 0.001) and groups B and C (MD = −0.446, *p* = 0.001), while the difference between groups A and C was not statistically significant (*p* = 0.215). Similarly, intention scores differed significantly between groups A and C (MD = −0.324, *p* < 0.001) and groups B and C (MD = −0.194, *p* = 0.001), with no significant difference observed between groups A and B (*p* = 0.079). Overall, participants receiving digital leaflet-based and educational video-based genetic counseling demonstrated more favorable improvements in knowledge and intention outcomes compared with those receiving direct face-to-face counseling. These patterns of change across groups and measurement times are further illustrated in Figure 3.

### 3.4. Sensitivity Analysis

Table 5 presents the results of the adjusted Generalized Estimating Equations (GEEs) sensitivity analysis conducted to account for baseline differences among intervention groups. The model included intervention group, time, and group-by-time interaction terms, with adjustment for sex, employment status, and monthly income. The results showed significant group-by-time interaction effects after adjustment, with knowledge significantly affected in Group C (β = −2.719, *p* < 0.001), attitude significantly affected in Group B (β = 0.842, *p* < 0.001), and screening intention showing significant effects in both Group B (β = −0.956, *p* < 0.001) and Group C (β = −0.788, *p* < 0.001). These findings indicate that the observed intervention effects remained statistically significant even after controlling for potential confounding sociodemographic variables, thereby confirming the robustness of the main study results.

## 4. Discussion

### 4.1. Comparative Effectiveness of Digital and Conventional Genetic Counseling Modalities

This study demonstrated that digital genetic counseling modalities were more effective than conventional face-to-face counseling in improving knowledge, attitudes, and premarital screening intentions related to thalassemia prevention among prospective couples. Digital leaflet-based counseling produced the greatest improvements in knowledge and screening intention, whereas educational video-based counseling significantly improved all measured outcomes, particularly attitudes. In contrast, direct face-to-face counseling did not produce statistically significant changes across the measured outcomes. These findings indicate that digitally delivered counseling may offer greater effectiveness in promoting preventive awareness and decision-making among reproductive-age populations.

The present findings are consistent with previous evidence demonstrating the advantages of digital genetic counseling in improving educational and psychosocial outcomes. Telegenetics has been shown to significantly improve fulfillment of patient expectations and perceived personal control compared with conventional counseling [28]. Similarly, educational video interventions have demonstrated superior effects on genetic knowledge acquisition, risk perception, and willingness to participate in screening compared with text-based educational approaches [29]. Supporting these findings, a recent meta-analysis reported that digital tools significantly increased completion of pretest counseling processes compared with standard approaches while producing knowledge outcomes comparable to in-person counseling [30]. Collectively, these findings suggest that digital delivery can support both cognitive understanding and behavioral readiness toward preventive genetic screening.

Several mechanisms may explain the superiority of digital interventions observed in this study. First, digital delivery ensures standardized and consistent educational exposure across participants, minimizing variability in communication quality that commonly occurs during interpersonal counseling. Second, digital platforms enable repeated access and self-paced learning, allowing participants to revisit complex concepts such as autosomal recessive inheritance, carrier status, and reproductive risk. This repeated exposure likely reinforces comprehension and strengthens preventive decision-making. In addition, educational videos may enhance attitudinal change through simultaneous visual and auditory stimulation, thereby increasing emotional engagement and facilitating deeper processing of health information through multimedia learning mechanisms [29].

By contrast, the limited effectiveness of face-to-face counseling observed in this study may reflect practical and contextual limitations. One-time counseling sessions may provide insufficient reinforcement for retention of complex genetic information, particularly among participants with limited prior exposure to hereditary health concepts [31]. Furthermore, interpersonal counseling effectiveness may vary according to communication style, educator competency, and participant willingness to openly discuss sensitive issues related to reproductive risk and hereditary disease. Such variability may reduce intervention consistency and weaken educational impact.

Digital interventions may also offer practical advantages that further support participant engagement. Previous studies have shown that digital counseling substantially reduces direct clinical interaction time while maintaining high patient satisfaction and lowering delivery costs compared with conventional face-to-face counseling [32,33,34,35]. Greater convenience, privacy, and flexibility may encourage participants to engage more actively with educational content and reflect more carefully on preventive decisions.

### 4.2. Behavioral and Sociocultural Considerations in Premarital Thalassemia Screening

The present findings should be interpreted within broader behavioral and sociocultural contexts that influence premarital thalassemia screening decisions. Although digital counseling interventions improved participants’ knowledge, attitudes, and intentions, preventive health behavior is shaped not only by educational exposure but also by emotional, familial, cultural, and structural influences. Premarital screening for hereditary diseases is often associated with concerns regarding marriage suitability, family acceptance, social stigma, and uncertainty about future reproductive outcomes. Consequently, individuals may recognize the preventive importance of screening while simultaneously experiencing hesitation toward undergoing testing or acting upon screening results.

This discrepancy between knowledge acquisition and behavioral response has been consistently reported in previous studies. A previous study demonstrated that educational interventions significantly improved thalassemia-related knowledge, whereas behavioral outcomes such as screening uptake and reproductive decision-making remained inconsistent because of persistent sociocultural barriers [36]. Similar findings were observed in an Indonesian pilot study involving prospective couples, where knowledge scores improved significantly following the intervention, but changes in attitudes and behavioral intentions were relatively limited [37]. These findings suggest that increased knowledge alone may be insufficient to modify deeply rooted beliefs, emotions, and social considerations surrounding hereditary disease prevention.

In many Southeast Asian communities, marriage decisions are closely connected to broader family and societal expectations. Being identified as a thalassemia carrier may evoke fears of social judgment, relationship disruption, diminished marital prospects, or family disapproval. Such concerns may discourage individuals from participating in screening programs despite understanding their health benefits. Research among migrant populations in Thailand further reported that religious beliefs, sociocultural norms, and negative perceptions toward pregnancy termination constituted important barriers to thalassemia screening participation [38]. In addition, inadequate family support, negative cultural attitudes, and poor communication with healthcare providers have been identified as major barriers influencing screening acceptance [7]. Family pressure may also play a substantial role, particularly in contexts where postponing or canceling marriage arrangements after screening is considered socially unacceptable [39]. Collectively, these sociocultural dynamics may partly explain why improvements in knowledge do not always translate into proportional changes in attitudes or actual preventive behavior.

### 4.3. Implications for Reproductive Health and Premarital Screening Programs

The findings of this study highlight the potential role of digital genetic counseling in strengthening premarital thalassemia prevention programs, particularly in settings with limited healthcare resources. Compared with conventional face-to-face approaches, digitally delivered counseling demonstrated greater effectiveness in improving preventive awareness and screening-related decision-making, indicating its potential as a scalable strategy for reproductive health education [30]. Educational videos and digital leaflets may facilitate more standardized information delivery while expanding access to counseling services across community-based healthcare settings [32,33]. In addition, the flexibility and relative privacy offered by digital platforms may encourage greater engagement with sensitive issues related to carrier status, reproductive risk, and marital planning [34,35].

Nevertheless, improvements in knowledge and intention alone may not necessarily translate into actual screening behavior. Previous evidence suggests that sociocultural, emotional, and structural barriers continue to influence premarital thalassemia screening participation despite educational gains [7,36]. Therefore, future prevention programs should integrate digital education with culturally responsive counseling, family-centered communication, and supportive referral systems to strengthen informed reproductive decision-making and improve long-term screening uptake among prospective couples.

### 4.4. Strengths and Limitations

This study possesses several important strengths. First, to our knowledge, this is among the first multi-site three-arm randomized controlled trials comparing multiple genetic counseling modalities for thalassemia prevention among prospective couples. The randomized design strengthens causal inference and reduces selection bias in evaluating intervention effectiveness. Second, the multi-site approach involving 15 Religious Affairs Offices improves ecological validity and enhances the applicability of findings to real-world premarital educational settings. Third, the inclusion of multiple outcomes, including knowledge, attitudes, and screening intentions, provides a more comprehensive understanding of counseling effectiveness beyond cognitive outcomes alone. Finally, the use of standardized educational content across intervention groups improved intervention consistency and strengthened comparative validity.

Despite these strengths, several limitations should be acknowledged. First, unequal attrition across intervention groups, particularly the relatively high dropout rate in the digital leaflet group, may have introduced attrition bias and influenced the comparability of post-intervention outcomes. Second, significant baseline differences in several participant characteristics and outcome measures were observed despite computer-generated randomization. Although sensitivity analyses using adjusted Generalized Estimating Equations (GEE) yielded findings consistent with the primary analyses, residual confounding cannot be completely excluded. Third, the knowledge and attitude questionnaire demonstrated moderate internal consistency (Cronbach’s alpha = 0.603). In addition, attitude was assessed using three dichotomous (Yes/No) items, which may have limited its sensitivity to detect subtle changes and contributed to conservative effect size estimates; therefore, findings related to the attitude outcome should be interpreted with appropriate caution. Fourth, outcomes were assessed shortly after intervention delivery, precluding evaluation of their long-term sustainability. In addition, screening intention rather than actual screening behavior was measured, and intention was assessed using a single-item instrument adapted from previous validated research. Although this approach minimized respondent burden, it may have been less sensitive to capturing the multidimensional nature of behavioral intention. Finally, eligibility required access to a smartphone or digital device to ensure standardized delivery of all randomized interventions, which may limit the generalizability of the findings. Moreover, participants in the digital intervention groups were able to revisit the educational materials after the intervention, whereas the face-to-face group received a single counseling session. This difference in exposure intensity may have partially contributed to the greater improvements observed in the digital intervention groups. Future studies should incorporate longer follow-up periods, objective measures of screening behavior, psychometrically stronger attitude instruments, broader geographic settings, and strategies to standardize intervention exposure across different delivery modalities.

## 5. Conclusions

This multi-site three-arm randomized controlled trial found that digitally delivered genetic counseling modalities were associated with greater improvements in knowledge, attitudes, and intentions toward premarital thalassemia screening than conventional face-to-face counseling within the study setting. These findings should be interpreted in light of the study’s methodological limitations, including baseline imbalances, differential attrition, moderate reliability of some outcome measures, and the short follow-up period.

## Figures and Tables

**Table 1:** Summary of the intervention.

Components	Digital Leaflet	Educational Video	Face-to-Face
Educational content	Standardized thalassemia genetic counseling module	Same module	Same module
Delivery mode	Smartphone (PDF leaflet)	Smartphone (video)	Direct counseling
Exposure duration	Participants were instructed to read the leaflet for approximately 15–20 min.	Participants viewed the complete educational video (10–15 min)	A single counseling session lasting approximately 20–30 min.
Counselor qualification	Self-directed	Self-directed	Registered nurse trained in genetic counseling
Fidelity	Standardized leaflet	Standardized video	Standardized counseling script

**Table 2:** Sociodemographic characteristics of the participants.

Variables	Group 1 *n* = 41	Group 2 *n* = 53	Group 3 *n* = 59	Total	Homogeneity	
	f (%)	f (%)	f (%)		r	*p*-Value
**Age**						
≥mean (23.4)	19 (12.4)	21 (13.7)	30 (19.6)	70 (45.8)	0.045	0.578
<mean (23.4)	22 (14.4)	32 (20.9)	29 (19.0)	83 (54.2)		
**Sex**						
Male	12 (7.8)	18 (11.8)	29 (19.0)	59 (40.6)	0.169	0.037
Female	29 (19.0)	35 (22.9)	30 (19.6)	94 (61.4)		
**Education**						
Senior High School	27 (65.9)	30 (56.6)	35 (59.3)	92 (60.1)	0.047	0.563
University	14 (34.1)	23 (43.4)	24 (40.7)	61 (39.9)		
**Employment Status**						
Employed	14 (9.2)	23 (15.0)	24 (15.7)	61 (39.9)	0.349	<0.001 *
Unemployed	27 (17.6)	30 (19.6)	35 (22.9)	92 (60.1)		
**Take Home Pay**						
≥US$127	1 (0.7)	35 (22.9)	24 (15.7)	60 (39.2)	0.267	<0.001 *
<US$127	40 (26.1)	18 (11.8)	35 (22.9)	91 (60.8)		
**Knowledge**						
Good	3 (7.3)	14 (26.4)	48 (81.4)	65 (42.5)	0.617	<0.001 *
Poor	38 (92.7)	39 (73.6)	11 (18.6)	88 (57.5)		
**Attitude**						
Positive	40 (97.6)	15 (28.3)	41 (69.5)	96 (62.7)	0.174	0.031 **
Negative	1 (2.4)	38 (71.7)	18 (30.5)	57 (37.3)		
**Intention**						
Positive	3 (7.3)	43 (81.1)	51 (86.4)	97 (63.4)	0.621	<0.001 *
Negative	38 (92.7)	10 (18.9)	8 (13.6)	56 (36.6)		

Data are presented as frequency (f) and percentage (%). Homogeneity was tested using Pearson’s chi-square test. r indicates the contingency coefficient. * *p* < 0.001; ** *p* < 0.05.

**Table 3:** Within-group effects of the intervention on participants’ outcomes.

Variables	Group	Scores Changes			Z	r	*p*-Value
		Negative f (*n*)	Positive f (*n*)	Ties f (*n*)			
Knowledge	A	0 (0.0)	35 (85.4)	6 (14.6)	−5.916	0.92	<0.001 *
	B	1 (1.9)	33 (62.3)	19 (35.8)	−5.488	0.75	<0.001 *
	C	6 (10.2)	10 (16.9)	43 (72.9)	−1.000	0.13	0.317
Attitude	A	5 (12.2)	1 (2.4)	35 (85.4)	−1.633	0.25	0.102
	B	3 (5.7)	22 (41.5)	28 (52.8)	−3.800	0.52	<0.001 *
	C	13 (22.0)	15 (25.4)	31 (52.5)	−0.378	0.05	0.705
Intention	A	0 (0.0)	38 (92.7)	3 (7.3)	−6.164	0.96	<0.001 *
	B	17 (32.1)	4 (7.5)	32 (60.4)	−2.837	0.39	0.005 **
	C	3 (5.1)	4 (6.8)	52 (88.1)	−0.378	0.05	0.705

A = Digital leaflet-based genetic counseling (*n* = 41); B = educational video-based genetic counseling (*n* = 53); C = direct face-to-face genetic counseling (*n* = 59). Negative, positive, and ties indicate decreased, increased, and unchanged post-intervention scores, respectively. Z = Wilcoxon signed-rank test statistic; r = effect size. Statistical significance was set at *p* < 0.05. * *p* < 0.001; ** *p* < 0.05.

**Table 4:** Between-group effects of the intervention on participants’ outcomes.

Variables	Group	MD	SE	t	*p*_Tukey	η^2^	*p*-Value
Knowledge	A–B	−0.110	0.233	−0.475	0.883	0.059	<0.001 *
	A–C	−1.047	0.227	−4.603	<0.001		
	B–C	−0.937	0.221	−4.442	<0.001		
Attitude	A–B	0.691	0.138	5.010	<0.001	0.080	<0.001 *
	A–C	0.244	0.135	1.810	0.215		
	B–C	−0.446	0.125	−3.560	0.001		
Intention	A–B	−0.130	0.058	−2.240	0.079	0.084	<0.001 *
	A–C	−0.324	0.057	−5.700	<0.001		
	B–C	−0.194	0.053	−3.660	0.001		

A = Digital leaflet-based genetic counseling (*n* = 41); B = educational video-based genetic counseling (*n* = 53); C = direct face-to-face genetic counseling (*n* = 59). MD = mean difference; SE = standard error; η^2^ = eta squared effect size. *p*_Tukey values were obtained using Tukey post hoc comparisons. Statistical significance was set at *p* < 0.05. * *p* < 0.001.

**Table 5:** Sensitivity analysis.

Variables	Parameter	β	SE	Wald χ^2^	95% CI (Lower–Upper)	*p*-Value
Knowledge	Time	3.024	0.294	106.06	(2.449)–(3.600)	<0.001 *
	Group B × Time	−0.647	0.452	2.05	(−1.532)–(0.238)	0.152
	Group C × Time	−2.719	0.411	43.87	(−3.524)–(−1.915)	<0.001 *
Attitude	Time	−0.220	0.156	1.97	(−0.526)–(0.087)	0.160
	Group B × Time	0.842	0.238	12.53	(0.376)–(1.309)	<0.001 *
	Group C × Time	0.304	0.245	1.54	(−0.177)–(0.785)	0.215
Intention	Time	0.805	0.079	104.32	(0.650)–(0.959)	<0.001 *
	Group B × Time	−0.956	0.117	67.12	(−1.185)–(−0.727)	<0.001 *
	Group C × Time	−0.788	0.091	75.56	(−0.966)–(-0.610)	<0.001 *

Group A = Digital leaflet-based genetic counseling; Group B = educational video-based genetic counseling; Group C = direct face-to-face genetic counseling. Models were adjusted for sex, employment status, and monthly income. Group A served as the reference category. β = Regression coefficient; SE = sandard error; Wald χ^2^ = significance of the parameter estimates. Significant Group × Time interaction terms indicate differential changes in outcomes over time according to counseling modality. Statistical significance was set at *p* < 0.05. * *p* < 0.001.

## Data Availability

The data presented in this study are available on request from the corresponding author due to (specify the reason for the restriction).

## Abbreviations

The following abbreviations are used in this manuscript:

ANOVA	Analysis of Variance
CONSORT	Consolidated Standards of Reporting Trials
GEE	Generalized Estimating Equations
ITT	Intention-to-Treat
RCT	Randomized Controlled Trial
